# Hyaluronic acid/doxorubicin nanoassembly-releasing microspheres for the transarterial chemoembolization of a liver tumor

**DOI:** 10.1080/10717544.2018.1480673

**Published:** 2018-06-17

**Authors:** Song Yi Lee, Jin Woo Choi, Jae-Young Lee, Dae-Duk Kim, Hyo-Cheol Kim, Hyun-Jong Cho

**Affiliations:** a College of Pharmacy, Kangwon National University, Chuncheon, Gangwon, Republic of Korea;; b Department of Radiology, Seoul National University Hospital, Seoul National University College of Medicine, Seoul, Republic of Korea;; c College of Pharmacy, Chungnam National University, Daejeon, Republic of Korea;; d College of Pharmacy and Research Institute of Pharmaceutical Sciences, Seoul National University, Seoul, Republic of Korea

**Keywords:** Doxorubicin, liver tumor, microsphere, nanoassembly, transarterial chemoembolization

## Abstract

Doxorubicin (DOX)-loaded, hyaluronic acid-ceramide (HACE) nanoassembly-releasing poly(lactic-co-glycolic acid) (PLGA) microspheres (MSs) were developed for transarterial chemoembolization (TACE) therapy of liver cancer. DOX/HACE MSs with a mean diameter of 27 μm and a spherical shape were prepared based on the modified emulsification method. Their *in vitro* biodegradability in artificial biological fluids was observed. A more sustained drug release pattern was observed from DOX/HACE MS than from DOX MS at pH 7.4. The cellular internalization efficiency of DOX of the DOX/HACE MS group was higher than that of the DOX MS group in liver cancer cells (HepG2 and McA-RH7777 cells), mainly due to CD44 receptor-mediated endocytosis of the released DOX/HACE nanoassembly. In both HepG2 and McA-RH7777 cells, the antiproliferation and apoptotic potentials of the DOX/HACE MS were significantly higher than those of the DOX MS (*p* < .05). Notably, in the McA-RH7777 tumor-implanted rat models, a better tumor growth suppression, a lower tumor viable portion, and a higher incidence of apoptosis were presented in the DOX/HACE MS group than in the DOX MS group after intra-arterial (IA) administration. DOX/HACE-based nanoassembly release from the DOX/HACE MS seems to elevate the cellular accumulation of DOX and its anticancer activities. The developed DOX/HACE MS can be used as a drug-loaded HA nanoassembly-releasing MS system for TACE therapy of liver cancer.

## Introduction

The complete remission of hepatocellular carcinoma (HCC) is known to be difficult due to disease heterogeneity and its tendencies toward metastasis and recurrence (Dutta & Mahato, 2017). Various therapeutic approaches (e.g. liver transplantation, surgical resection, local ablation, transarterial chemoembolization (TACE), radiotherapy, and molecular-targeted agents) have been used for the therapy of HCC (Genco et al., 2013; Grandhi et al., 2016; Dutta & Mahato, 2017; Kim, 2017; Lee & Khan, 2017). The application of surgical resection and local ablation has been restricted for patients with large/multiple tumors, extrahepatic spread of HCC, and decreased hepatic function. In this context, TACE is widely applied to those patients with a minimal invasiveness. TACE can induce tumor necrosis and control tumor growth while conserving healthy liver tissues as much as possible (Ramsey et al., 2002). However, there still exist controversies regarding the usefulness of chemotherapy over embolization, considering the toxicity of chemotherapeutic agents (Pleguezuelo et al., 2008; Brown et al., 2016; Lanza et al., 2016). Considering the angiogenesis induced by hypoxia following TACE, more elaborate therapeutic approaches are necessary for elevating the anticancer efficacies of TACE and minimizing its toxicities.

Conventional TACE (cTACE) has been regarded as a gold standard for the treatment of intermediate-stage HCC (Nishikawa et al., 2014). In cTACE therapy, chemotherapeutic drugs and embolic agents can be administered to the feeding artery of the tumors through a catheter (Choi et al., 2014; Nishikawa et al., 2014). Lipiodol, an oil-based contrast medium, can be injected into the artery as an emulsion formulation with hydrophilic or hydrophobic chemotherapeutic drugs (Idée & Guiu, 2013). In a drug-eluting bead (DEB)-TACE method, the application of embolic microspheres (MSs) charged with cytotoxic agents to the hepatic artery can provide simultaneous drug-releasing and embolization functions (Facciorusso, 2018). The doxorubicin (DOX)-bound poly(vinyl alcohol) (PVA)-based bead (i.e. DC bead) has also been used clinically for the TACE of HCC (Gao et al., 2013; Lewis & Holden, 2011).

Recently, MS formulations composed of poly(lactic-co-glycolic acid) (PLGA), a biodegradable polymer, have been developed for the therapy of HCC (Choi et al., 2015; Liang et al., 2017). In our previous study (Choi et al., 2015), DOX-loaded PLGA MSs were fabricated by the solid-in-oil-in-water method, and its TACE application for HCC via an intra-arterial (IA) route was investigated. DOX was successfully incorporated into the PLGA MS, and its biodegradability and therapeutic efficacies were identified. In this investigation, receptor-mediated endocytosis of DOX for efficient cellular internalization was introduced into the MS formulation. Various types of nanoparticles have been designed to improve tumor targeting capability and cancer therapeutic efficacies (Jia et al., 2013; Peng et al., 2015). To reduce toxicity of nanocarrier itself, biocompatible and biodegradable materials have been introduced to deliver therapeutic cargos (Du et al., 2014; Li et al., 2014; Cao et al., 2015; Wang et al., 2015; Zhang et al., 2016b; Hu et al., 2017b). Among them, hyaluronic acid (HA)-based nanoparticles have been widely investigated as one of the active tumor targeting strategies via CD44 receptor-mediated endocytosis (Zheng et al., 2016a; Hu et al., 2017a). By entrapping the composite of DOX and hyaluronic acid-ceramide (HACE) in the PLGA MS, the DOX/HACE nanoassembly may be released from the MS after its administration into the hepatic artery. The release of the DOX/HACE nanoassembly from the MS into the closed intravascular space with the tumor mass may lead to improved cellular uptake and anticancer activities. To the best of our knowledge, this is the first report of a drug-loaded HA nanoassembly-releasing PLGA MS formulation; furthermore, we systemically evaluated its therapeutic potentials for HCC.

## Materials and methods

### Materials

DOX HCl was purchased from Boryung Pharmaceutical Co., Ltd. (Seoul, Korea). Chloromethylbenzoyl chloride, PLGA (lactide:glycolide = 75:25, 66–107 kDa molecular weight (MW)), PVA (MW: 30–70 kDa), and tetra-*n*-butylammonium hydroxide (TBA) were supplied by Sigma-Aldrich Co. (St. Louis, MO, USA). 2,3,5–Triiodobenzoic acid (TIBA) was purchased from Tokyo Chemical Industry Co., Ltd. (Tokyo, Japan). HA oligomer (MW: 4–8 kDa) and DS-Y30 (ceramide 3B; mainly *N*-oleoylphytosphingosine) were provided by SK Bioland Co., Ltd. (Cheonan, Republic of Korea) and Doosan Biotech Co., Ltd. (Yongin, Republic of Korea), respectively. Dulbeco’s modified Eagle’s media (DMEM), RPMI1640 (developed by Roswell Park Memorial Institute), fetal bovine serum (FBS), penicillin, and streptomycin were purchased from Gibco Life Technologies, Inc. (Grand Island, NY, USA). All other reagents were of analytical grade and were acquired from commercial sources.

### Preparation and characterization of DOX-loaded MSs

DOX-loaded MSs were fabricated using a modified emulsification method (Choi et al., 2015). In this study, a DOX base was incorporated into MS formulations by first dissolving DOX HCl (100 mg) in dimethyl sulfoxide (DMSO; 10 mL) and triethylamine (0.12 mL). After stirring for 12 h, the resulting solution was lyophilized (Cho et al., 2012).

DOX MS consisted of DOX (chemotherapeutic agent), PLGA (polymer matrix), and TIBA (CT contrasting agent). PLGA (75 mg) and TIBA (30 mg) were dissolved in dichloromethane (1.5 mL). DOX base (7.5 mg) dissolved in DMSO (75 μL) was added to the solution of PLGA and TIBA and they were mixed to homogeneity. This organic phase was put into the 2% PVA solution (30 mL) and that oil-in-water emulsion was mixed with a homogenizer at 9500 rpm for 10 sec. It was stirred for 2 h at room temperature to remove the organic solvents. After centrifuging at 10,000×*g* for 1 min, the MS pellet was collected. The MS pellet was resuspended in distilled water (DW) and then lyophilized.

In the DOX/HACE MS, HACE was incorporated into the PLGA MS to release the nanoassembly of DOX. HACE was synthesized according to the previously reported method (Cho et al., 2011). Briefly, HA (12.21 mmol) and TBA (9.77 mmol) were dispersed in double-distilled water (DDW, 60 mL) by stirring for 30 min, and activated HA-TBA was obtained by freeze-drying. DS-Y30 ceramide (8.59 mmol) and triethylamine (9.45 mmol) in tetrahydrofuran (THF, 25 mL) and 4-chloromethylbenzoyl chloride (8.59 mmol) in THF (10 mL) were blended to make the DS-Y30 linker. That mixture was stirred for 6 h at 60 °C and the DS-Y30 linker was obtained by concentration and recrystallization. HA-TBA (8.10 mmol) and the DS-Y30 linker (0.41 mmol) were solubilized in the mixture of THF and acetonitrile (4:1, v/v) and the solution was stirred for 5 h at 40 °C. Purified HACE was acquired by removing impurities and organic solvents.

HACE (7.5 mg) dissolved in methanol (925 μL) and DOX (7.5 mg) in DMSO (75 μL) were blended. A 2% PVA solution (75 μL) was added to that mixture. PLGA (75 mg) and TIBA (30 mg) were dissolved in dichloromethane (1.5 mL), and the solution was mixed with DMSO (45 μL). The PLGA/TIBA solution was then mixed with the HACE/DOX solution. This mixture was added to the 2% PVA solution (29.925 mL), and that emulsion was blended with a homogenizer at 9500 rpm for 10 sec. That emulsion was stirred for 2 h at room temperature to eliminate the organic solvents. After centrifuging for 1 min, the pellet of MS was obtained. The pellet of MS was resuspended in DW, and it was then freeze-dried.

The particle size of the DOX MS and DOX/HACE MS was measured using a laser diffraction particle size analyzer (Microtrac S3500, Microtrac Inc., Montgomeryville, PA, USA) according to the manufacturer’s instructions. The fluorescence signals of DOX, implying the intraparticle distribution of the drug in the MS, were observed by confocal laser scanning microscopy (CLSM) (LSM 710; Carl-Zeiss, Thornwood, NY, USA). The content of DOX in the MSs was quantitatively analyzed by high-performance liquid chromatography (HPLC) as previously reported (Choi et al., 2015), using a Waters HPLC system (Waters Co., Milford, MA, USA) equipped with a separation module (Waters e2695), a fluorescence detector (Waters 2475), and a column (reverse phase, C18, 250 × 4.6 mm, 5 μm; Xbridge, Waters Co.). The mobile phase was a mixture of 10 mM potassium phosphate buffer (pH 2.5) and acetonitrile (including 0.1% triethylamine) (73:27, v/v). The flow rate was set as 1 mL/min, and the injection volume was 20 μL. The fluorescence signal of DOX was measured at 480 nm (excitation) and 560 nm (emission) wavelengths. The lower limit of quantification (LLOQ) of DOX was 25 ng/mL in this assay. The precision and accuracy of the established method were within the acceptable range. The content of iodine in MSs was quantitatively determined by inductively coupled plasma-optical emission spectroscopy (ICP-OES, 730-ES, Agilent Technologies, Santa Clara, CA, USA). The location of DOX in the DOX MS and the DOX/HACE MS was observed by CLSM (LSM 710, Carl-Zeiss, Thornwood, NY, USA).

The particle properties of released materials from the DOX-loaded MSs were investigated by a dynamic light scattering (DLS) method and field emission-transmission electron microscopy (FE-TEM) imaging. DOX MS and DOX/HACE MS containing 100 μg DOX were dispersed in DW (1 mL) and were then incubated at 37 °C for 7 days. After centrifuging the dispersion samples, the particle size and polydispersity index values of the supernatant were measured by the DLS method (ELS-Z1000; Otsuka Electronics, Tokyo, Japan) according to the manufacturer’s protocol. For TEM imaging, an aliquot of specimen was stained with 2% (w/v) phosphotungstic acid and then placed on copper grids with films. Thereafter, it was dried for 10 min and observed by FE-TEM (JEM 2100F; JEOL, Tokyo, Japan).

### *In vitro* degradation study of MS

The *in vitro* degradability of DOX MS and DOX/HACE MS was assessed in artificial biological fluids. MSs were suspended in 50% (v/v) FBS or phosphate-buffered saline (PBS) (pH: 7.4) and then incubated at 37 °C for 14 days. MSs in each medium were centrifuged at 16,100×*g* for 5 min and the pellet of MSs was freeze-dried. MSs were sputter-coated with gold, and their shape was observed by a field emission-scanning electron microscope (FE-SEM; SUPRA 55VP, Carl Zeiss, Oberkochen, Germany).

### *In vitro* drug release test

The release profile of DOX from MSs was evaluated under different pH conditions. DOX MS or DOX/HACE MS, corresponding to approximately 75 μg of the drug, was suspended in DW (0.15 mL) and then placed into a mini GeBA-flex tube (14 kDa MW cutoff; Gene Bio-Application Ltd., Kfar Hanagide, Israel). Then, the tube was transferred to 2 mL of release medium (PBS; pH: 5.5, 6.8, and 7.4) and incubated in a shaking incubator at 37 °C and 50 rpm. At determined times (1, 2, 4, 6, 8, 24, 48, 72, 96, 120, 144, and 168 h), the MS-loaded tube was moved to the equivalent volume of fresh release medium. The released amounts of DOX were quantitatively determined by the previously described HPLC assay.

### Cellular uptake study

HepG2 and McA-RH7777 cells were purchased from the Korean Cell Line Bank (KCLB; Seoul, Republic of Korea) and the American Type Culture Collection (ATCC; Manassas, VA, USA), respectively. HepG2 cells were cultured with RPMI 1640 containing 10% (v/v) FBS and 1% (v/v) penicillin (100 U/mL) and streptomycin (0.1 mg/mL) at 37 °C in a humidified 5% CO_2_ atmosphere. McA-RH7777 cells were cultured with DMEM containing 10% (v/v) FBS and 1% (v/v) penicillin (100 U/mL) and streptomycin (0.1 mg/mL) under the same culture conditions. After obtaining 70%–80% confluency in a cell culture dish, cells (at a density of 6.0 × 10^5^ cells per well) were seeded onto six-well plates and then incubated for 1 day at 37 °C. DOX solution, DOX MS dispersion, and DOX/HACE MS dispersion (including 10 µg/mL DOX) were applied to the cells, and they were incubated for 2 and 6 h. Each sample was eliminated and cells were washed with PBS (pH: 7.4) at least thrice. After centrifuging, cell pellets were resuspended in PBS containing 2% FBS (v/v) prior to flow cytometry analysis. Cell counts based on fluorescence intensity were measured using a FACSCalibur fluorescence-activated cell sorter (FACS^TM^) equipped with CellQuest software (Becton Dickinson Biosciences, San Jose, CA, USA).

### *In vitro* anticancer activity tests

HepG2 and McA-RH7777 cells were cultured as described above. The antiproliferation efficacies of the DOX-loaded MSs were assessed by the colorimetric method using a tetrazolium compound [3-(4,5-dimethylthiazol-2-yl)-5-(3-carboxymethoxyphenyl)-2-(4-sulfophenyl)-2H-tetrazolium, inner salt; MTS] and an electron coupling reagent (phenazine ethosulfate; PES). Cells were seeded onto 96-well plates at a density of 5.0 × 10^3^ cells per well and were then incubated for 1 day. DOX solution, blank MS, DOX MS, blank HACE MS, and DOX/HACE MS with various concentrations (corresponding to 0.05, 0.1, 0.5, 1, 5, and 10 µg/mL concentrations of DOX) were applied to cells and incubated for 72 h at 37 °C. After eliminating each sample, CellTiter 96 Aqueous One Solution Cell Proliferation Assay Reagent (Promega Corp., Madison, WI, USA), including MTS and PES, was added to cells and treated according to the manufacturer’s instruction. The absorbance was measured at 490 nm using a multimode microplate reader (SpectraMax i3, Molecular Devices, Sunnyvale, CA, USA) and the cell viability (%) was calculated by comparing with that of the control group.

HepG2 and McA-RH7777 cells were cultured as described above. Cells were seeded onto six-well plates at a density of 1.0 × 10^5^ cells per well and were then incubated for 1 day at 37 °C. DOX solution, DOX MS, and DOX/HACE MS, at 1 μg/mL DOX, were applied to the cells and incubated for 24 h. Each sample was removed, and cells were washed with PBS (pH: 7.4) at least thrice. Cell pellets were obtained by centrifuging at 16,100×*g* for 5 min, and they were suspended in the reaction buffer of FITC Annexin V Apoptosis Detection Kit (BD Pharmingen, BD Biosciences, San Jose, CA, USA). According to the manufacturer’s protocol, cells were stained with Annexin V-fluorescein isothiocyanate (FITC) and propidium iodide (PI). Fluorescence intensity values in cells were analyzed by a FACSCalibur FACS^TM^ equipped with CellQuest software (Becton Dickinson Biosciences).

### *In vivo* antitumor efficacy tests

Male Sprague-Dawley (SD) rats (Orient Bio, Sungnam, Korea) with a body weight of approximately 450 g were used. They were reared in a light-controlled room at 22 ± 2 °C and at 55 ± 5% relative humidity. Animal experiments were performed according to the National Institutes of Health Guide for the Care and Use of Laboratory Animals (NIH Publications No. 8023, revised 1978). The protocols of animal experiments were approved by the institutional Animal Care and Use Committee (Seoul National University College of Medicine, Seoul National University Hospital).

McA-RH7777 cells were cultured as described above and 5.0 × 10^6^ cells in 50 μL serum-free DMEM were gently injected into the left lateral lobe of the liver in each SD rat (Choi et al., 2016). To minimize spontaneous tumor regression (Buijs et al., 2012), cyclosporine A (Chong Kun Dang Pharmaceutical Corp., Seoul, Korea) was subcutaneously injected at a dose of 20 mg/kg/day from 1 day before the tumor implantation to 4 days after surgery (Choi et al., 2016). Tumor induction in rats was identified by a clinical magnetic resonance (MR) scanner, and the rats were then randomly divided into three groups.

The IA administration of the MS formulation was performed by an experienced interventional radiologist according to published protocols (Choi et al., 2015, 2017). Under anesthesia via the intramuscular injection of zolazepam (5 mg/kg, Zoletil^®^; Virbac, Carros, France) and xylazine (10 mg/kg, Rompun^®^; Bayer-Schering Pharma, Berlin, Germany), a 1.6-French microcatheter (Nano 1.6; Create Medic, Yokohama, Japan) was inserted into the carotid artery. With the guidance from an X-ray fluoroscope, the catheter was inserted into the hepatic artery and MSs (DOX MS and DOX/HACE MS), suspended in the mixture of normal saline and iodine contrast agent (Ultravist 370; Bayer Healthcare, Leverkusen, Germany) at a DOX dose of 1 mg/kg, were infused via the microcatheter.

Alteration in the volume of liver tumor in control, DOX MS, and DOX/HACE MS groups was detected by clinical MR scanner on day 0, 3, and 7. MR image was obtained with a T2-weighted imaging sequence (bandwidth = 199 Hz/pixel; field of view = 80 × 65 mm; matrix = 256 × 177; repetition time/echo time = 4180/77 msec; slice thickness = 2 mm) (Choi et al., 2017). Tumor volume (*V*, mm^3^) was calculated with longer diameter (*a*, mm) and shorter diameter (*b*, mm) by the following formula:

$$V=\frac{4}{3}\pi \left(\frac{a}{2}\right){\left(\frac{b}{2}\right)}^{2}$$

Rats were sacrificed after 1 week, and liver, lung, spleen, kidney, and heart were dissected for histologic staining. Those specimens were fixed in a 4% (v/v) buffered formaldehyde solution and were subsequently embedded in paraffin. After deparaffinization and rehydration with a graded ethanol series, the specimens were stained with hematoxylin and eosin (H&E) and the liver tissue was further treated for a terminal deoxynucleotidyl transferase dUTP nick end labeling (TUNEL) assay by standard protocols. For TUNEL staining of liver tissue samples, the chromogen 3,3′-diaminobenzidine (DAB) was incubated with tissues for color development to detect deoxyribonucleic acid (DNA) fragmentation, an indication of apoptotic signaling cascades. The viable tumor portion in liver tissue was visually evaluated by a pathologist who was blinded to the treatment allocation.

### Statistical analyses

Statistical analyses of data were conducted using Student’s *t*-test and analysis of variance (ANOVA). Data are presented as the mean ± standard deviation (SD). A two-sided *p* value of <.05 was considered to indicate statistical significance.

## Results and discussion

### Preparation and characterization of DOX-loaded MSs

In this study, the HACE/DOX composite-embedded MS was fabricated using a modified emulsification method. In our previous studies (Choi et al., 2015, 2017), PLGA-based MSs were developed for the locoregional delivery of anticancer agents to a liver tumor via the IA route. For the efficient entrapment of anticancer drugs (i.e. DOX and sorafenib) in MSs, a modified emulsification method was used. Notably, in our recent study (Choi et al., 2017), TIBA was added to MSs as a CT imaging contrast agent to monitor the *in vivo* fate of MSs after their IA administration. Although the sustained release property of the drug from PLGA-based MSs has already been presented in previous studies (Choi et al., 2015, 2017), their selective uptake into liver cancer cells has not been investigated. The DOX/HACE composite and TIBA were incorporated into the PLGA MS in this study and the release of the DOX/HACE-based nanoassembly from MSs was expected. HACE has been synthesized as an amphiphilic material composed of HA (hydrophilic backbone) and ceramide (CE, hydrophobic residue) (Figure S1) (Cho et al., 2011). HACE exhibited self-assembly properties in the aqueous environment (Cho et al., 2011). A poorly water-soluble drug can be entrapped in the internal cavity of HACE-based nanoparticles, and when this occurs, they exhibit CD44 receptor-mediated endocytosis and *in vivo* tumor targeting (Cho et al., 2011; Cho et al., 2012; Jin et al., 2012; Park et al., 2014; Lee et al., 2016; Jeong et al., 2017; Lee et al., 2017). Therefore, we expected that a HACE-based nanoassembly can increase the cellular uptake efficiency into liver cancer cells via interactions between HA and the CD44 receptor.

**Figure 1.**
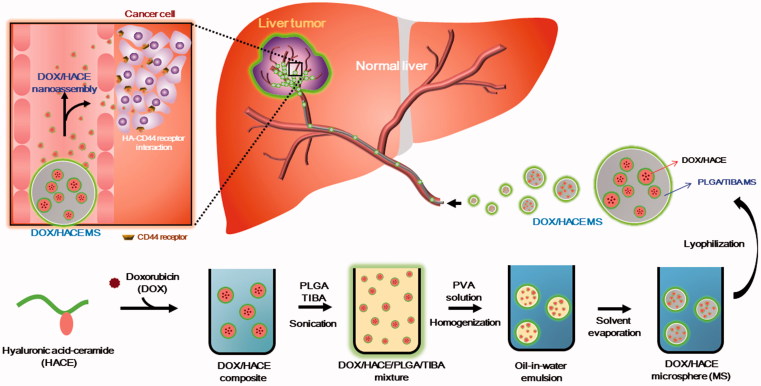
Scheme of the fabrication process of DOX/HACE MS.

As shown in Figure 1, DOX and HACE, dissolved in the mixture of methanol, DMSO, and PVA, were added to the organic phase (dichloromethane and DMSO) containing PLGA and TIBA. The combination produced an emulsion system. By evaporating the solvent, hardened MSs were obtained. DOX/HACE may be mainly located in the internal space of the MSs. After the administration of DOX/HACE MS into the hepatic artery, the biological fluids are expected to penetrate into the outer surface of MSs, and erosion and diffusion then act as major drug release mechanisms. Due to the amphiphilic property of HACE, it is expected that DOX/HACE will be released as a self-assembly structure in the adjacent aqueous environment.

**Figure 2.**
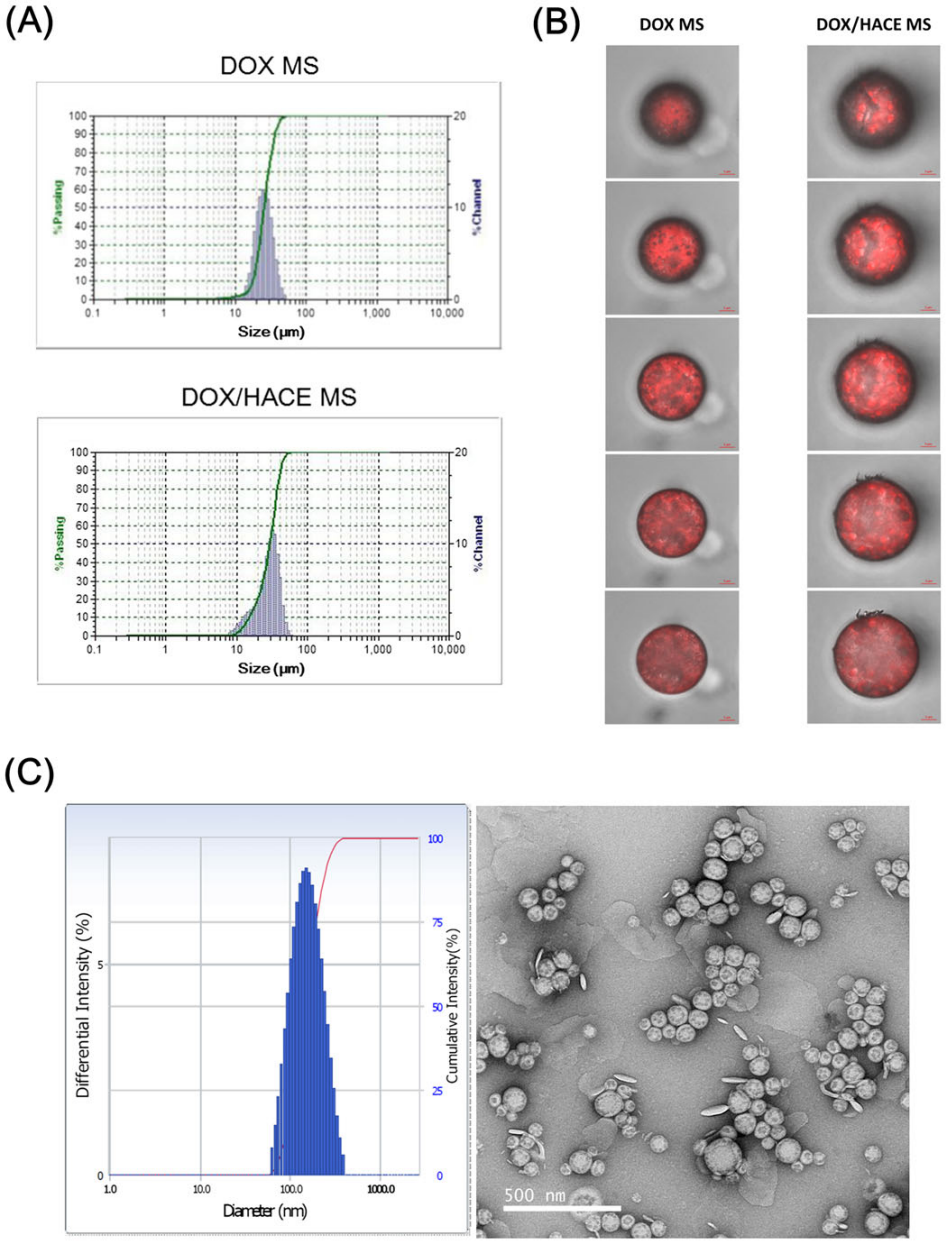
Particle characterization of DOX-loaded MS. (A) Size distribution profiles of DOX MS and DOX/HACE MS. (B) CLSM images of DOX MS and DOX/HACE MS. Depth-dependent cross-sectional images were observed by CLSM. Red fluorescence signal in MS indicates the intraparticle distribution of DOX. The length of the scale bar is 5 μm. (C) Particle characteristics of released DOX/HACE nanoassembly from DOX/HACE MS after incubating for 7 days. Size distribution profile (left) and TEM image (right) are presented. The length of the scale bar in the TEM image is 500 nm.

The mean diameters of DOX MS and DOX/HACE MS were 25 ± 3 μm and 27 ± 4 μm, respectively (Table S1 and Figure 2(A)). A unimodal particle size distribution was also observed (Figure 2(A)). According to the size distribution chart of the DOX MS group, the mean diameter of approximately 90% of MSs ranges from 16 to 40 μm. Also, the mean diameter of approximately 90% of DOX/HACE MS ranges from 13 to 44 μm. For efficient embolization of MSs in the hepatic artery, their size should fit within the internal diameters of the capillaries (8−10 µm) and terminal arterioles (10−50 µm) of the rat liver (Bao et al., 2006; Choi et al., 2017). In our previous studies (Choi et al., 2015, 2017), PLGA-based MSs with diameters of 20–30 μm exhibited sufficient embolization effect in the hepatic artery after their IA administration. The average values of encapsulation efficiency of DOX in the DOX MS and DOX/HACE MS groups were 97% and 65%, respectively (Table S1). The encapsulation efficiency values of DOX in MSs seem to be suitable for establishing an IA administration dose. The iodine contents (w/w%), measured by ICP-OES analysis, in DOX MS and DOX/HACE MS were 0.63 ± 0.03% and 0.71 ± 0.11%, respectively.

The distribution of DOX in MS was observed by CLSM imaging (Figure 2(B)). In the CLSM images of the DOX/HACE MS group, many dimples were observed, along with strong fluorescence signals in dimpled areas. The differences in the kind of organic solvents and the presence of HACE in the DOX/HACE MS group could be responsible for this morphological feature. The aggregates of DOX and HACE in those dimples may escape from MSs after dispersing in the aqueous environment. Differences in the intraparticle distribution of the drug may also affect drug release pattern from MSs.

In addition, the release of the DOX/HACE-based nanoassembly from DOX/HACE MS was verified by particle size analysis and TEM imaging (Figure 2(C)). After incubating DOX MS and DOX/HACE MS in DW for 7 days, the supernatant was analyzed. In the DOX MS group, microparticles with a broad size distribution were measured by the DLS method. In contrast, the hydrodynamic size and polydispersity index of the DOX/HACE MS group were 236 ± 49 nm and 0.16 ± 0.03, respectively. A round shape and the corresponding particle size were also observed by TEM imaging. These data support the conclusion that the DOX/HACE-based nanoassembly can be released from the DOX/HACE MS after incubating in the aqueous environment.

### *In vitro* degradation of MS

**Figure 3.**
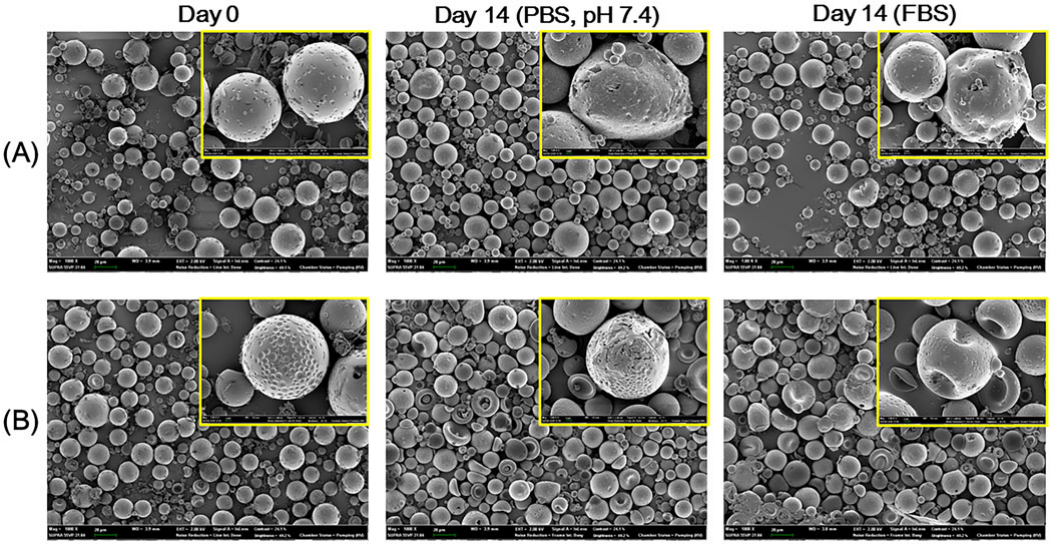
*In vitro* degradation test of fabricated MS. DOX MS (A) and DOX/HACE MS (B) were incubated in PBS (pH 7.4) and FBS for 14 days. The morphology of MS was observed by SEM (scale bar = 20 μm). Inset with the yellow boundary indicates the magnified image (scale bar = 2 μm).

The initial morphology of prepared MSs and their alteration after being suspended in the aqueous buffer (PBS) and serum media (50% FBS) were observed by FE-SEM imaging (Figure 3). The smooth texture of the outer surface and the round shape of the MSs were evident on day 0 (Figure 3). This implies that the developed MSs can be safely applied to embolize the hepatic artery without severe damage to the vascular endothelium. The biodegradation profiles of DOX MS and DOX/HACE MS in the blood stream were estimated by their *in vitro* stability test. The morphological changes of MSs were assessed by comparing the initial image to those in aqueous buffer and serum media on day 14. As observed in Figure 3, after incubating for 14 days in PBS and FBS (50%) media, MSs appeared irregularly shaped and had multiple pores. Notably, as shown in the CLSM images of MSs (Figure 2(B)), more dimples were presented in DOX/HACE MS group rather than DOX MS group (Figure 3). Strong fluorescence signals were shown in those dimples and they indicate the existence of DOX/HACE composite (Figure 2(B)). After incubating in PBS and FBS for 14 days, more significant surface erosion and rupture, which might be induced by the release of DOX/HACE composite, were observed in DOX/HACE MS group compared with PLGA MS group. The slight difference in the degradation patterns between DOX MS and DOX/HACE MS may be due to the incorporation of HACE and its contribution to drug release from the DOX/HACE MS. PLGA is known as a biodegradable material due to the cleavage of ester linkages that produce lactic acid and glycolic acid (Danhier et al., 2012). A sustained drug release may also be supported by the degradation of PLGA-based MSs. The observed morphological changes in MSs could be involved in their degradation kinetics in the blood stream.

### *In vitro* drug release

**Figure 4.**
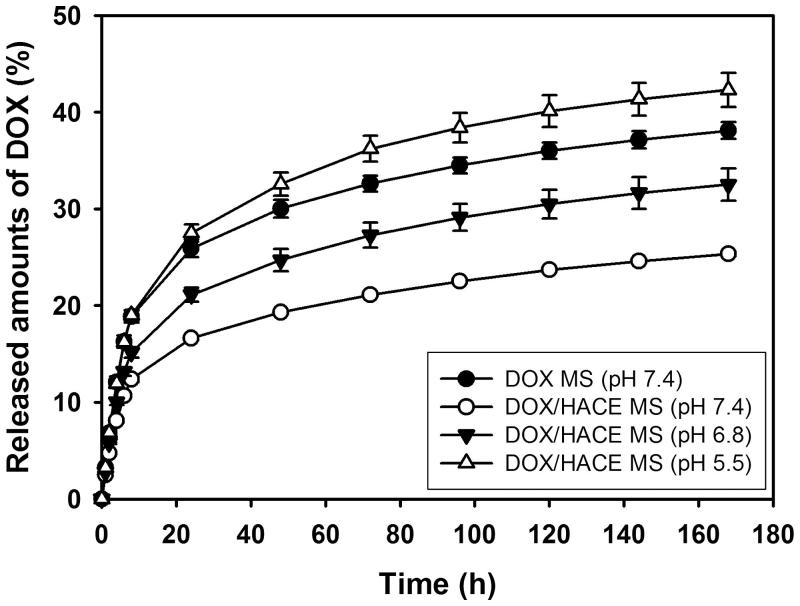
Drug release profiles from DOX MS (pH: 7.4) and DOX/HACE MS (pH: 7.4, 6.8, and 5.5). Each point indicates the mean ± SD (*n* = 3).

The DOX release profiles of the DOX MS and DOX/HACE MS groups were acquired after 7 days of incubation in the aqueous buffers with different pH values (Figure 4). Although the particle properties of nanoassembly were evaluated by DLS data and TEM image (Figure 2(C)), the accurate chemical composition (i.e. the weight ratio between DOX and HACE) of DOX/HACE nanoassembly cannot be determined. Therefore, DOX/HACE nanoassembly group was not included in drug release test due to the absence of drug content value. However, sustained drug release patterns (for several days) from HACE-based nanoparticles were already identified in our previous studies (Cho et al., 2012; Park et al., 2014; Lee et al., 2016), and these data could be applied to predict drug release profile from DOX/HACE nanoassembly in this study. The released amounts of DOX from DOX MS at pH 7.4 on day 1 and day 7 were 3.25 ± 0.24% and 38.10 ± 0.86%, respectively. In case of DOX/HACE MS, the released amounts of DOX at pH 7.4 on day 1 and day 7 were 2.51 ± 0.14% and 25.34 ± 0.26%, respectively. The presence of HACE and the difference in surface morphology of the MSs may retard drug release from the MS. The released amounts of DOX from DOX/HACE MS at pH 6.8 and pH 5.5 on day 7 were 32.53 ± 1.67% and 42.30 ± 1.78%, respectively. In more acidic pH, the amounts of DOX released are enhanced compared to normal physiological pH. As revealed in our previous study (Choi et al., 2015), this might be due to the improved DOX solubility in acidic pH and the reduction of interactions between drug and MS. In both MS formulations, sustained drug release for 1 week was observed, which may guarantee its suitability for TACE application. Of note, there was a difference in the amounts of drug released from DOX MS compared to our previous data (Choi et al., 2015). Different release test protocols and different formulation compositions (i.e. the existence of TIBA) may have affected the release profile of DOX from MS in this study.

### Cellular uptake

**Figure 5.**
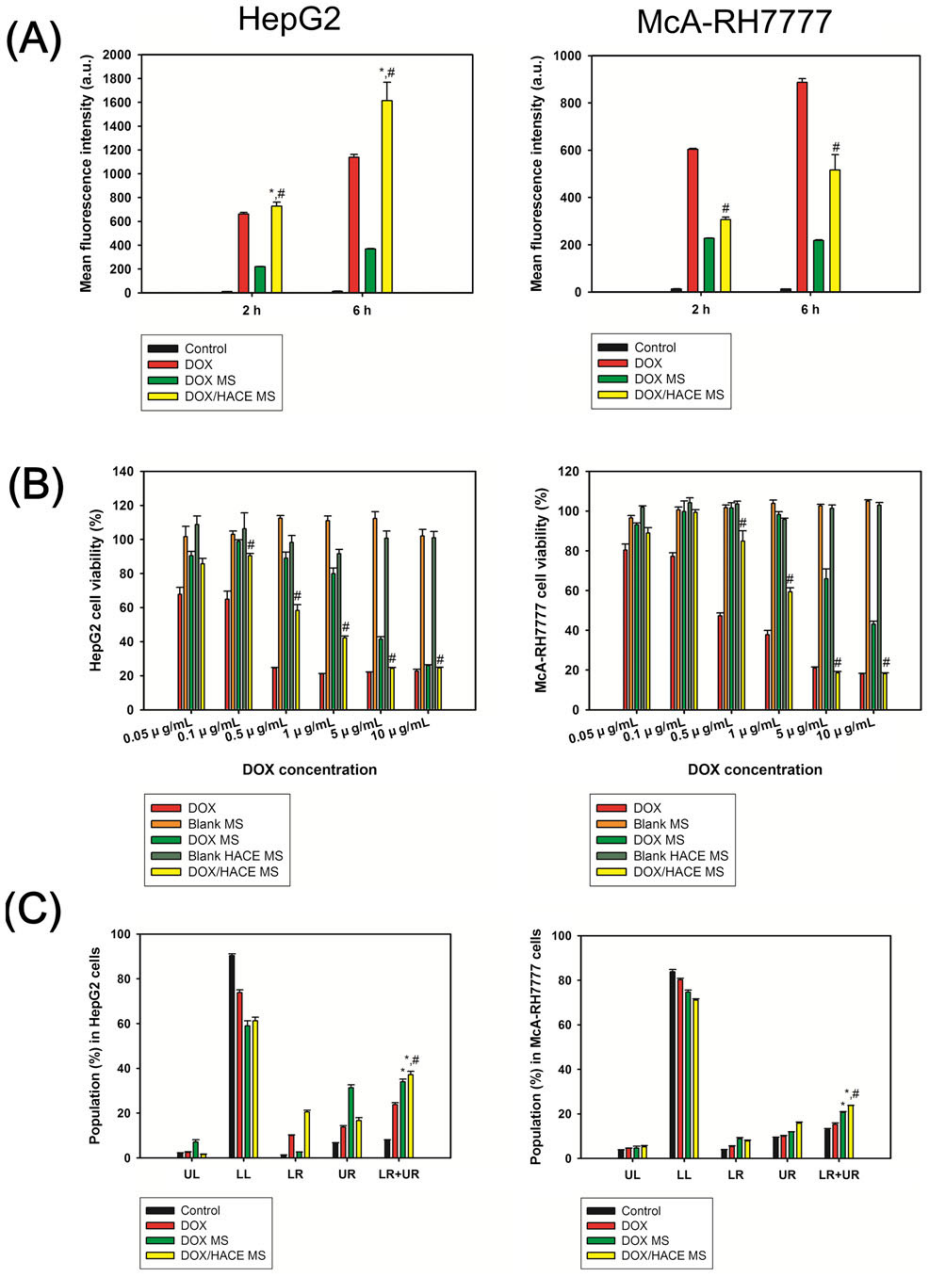
Cellular accumulation efficiency and *in vitro* anticancer activities in HepG2 and McA-RH7777 cells. (A) The mean fluorescence intensity values of DOX, measured by flow cytometry, in HepG2 cells and McA-RH7777 cells. Each point indicates the mean ± SD (*n* = 3). *p < .05, compared with DOX group. ^#^*p* < .05, compared with DOX MS group. (B) Antiproliferation efficacy, evaluated by MTS-based assay, of DOX-loaded MSs in HepG2 cells and McA-RH7777 cells. Cell viability (%) is presented as the relative percentage based on the control (no treatment) group. Each point indicates the mean ± SD (*n* = 3). ^#^*p* < .05, compared with DOX MS group. (C) Apoptotic efficacy of developed MS, measured by Annexin V and PI-based assays, in HepG2 cells and McA-RH7777 cells. UL, LL, LR, and UR indicate upper left, lower left, lower right, and upper right, respectively. Each point indicates the mean ± SD (*n* = 3). *p < .05, compared with DOX group. ^#^*p* < .05, compared with DOX MS group.

The cellular accumulation efficiency of DOX from DOX-loaded MS formulations was assessed in HepG2 and McA-RH7777 cells (Figure 5(A)). HepG2 and McA-RH7777 cells originated from human HCC and Buffalo rat Morris hepatoma 7777, respectively. HepG2 cell was chosen because it is a CD44 receptor-positive cell line (Wang et al., 2012), whereas McA-RH7777 cell was chosen because variant isoforms of CD44 (CD44v) (especially CD44v8–10), rather than other endogenous isoforms, are overexpressed (Lau et al., 2014). It is also reported that CD44v may have higher affinity to HA compared to the standard isoform of CD44 (CD44s) (Misra et al., 2011). Thus, these two types of cells can be used properly to evaluate CD44 receptor-mediated endocytosis.

Intracellular fluorescence signals, indicating the cellular uptake amount of DOX, were quantitatively analyzed by flow cytometry after incubating DOX, DOX MS, and DOX/HACE MS for 2 and 6 h in HepG2 and McA-RH7777 cells (Figure 5(A)). In HepG2 cells, the DOX/HACE MS group exhibited significantly improved cellular uptake of the drug compared to the DOX and DOX MS groups (*p* < .05). Notably, after 2 and 6 h of incubation, the mean fluorescence intensity values of the DOX/HACE MS group were 3.3- and 4.4-fold higher than those of the DOX MS group, respectively. Also in McA-RH7777 cells, the mean fluorescence intensity values of the DOX/HACE MS group were 1.3- and 2.4-fold higher than those of the DOX MS group after 2 and 6 h incubation, respectively (*p* < .05). Improved cellular accumulation of drug in the DOX/HACE MS group, compared with the DOX MS group, may be explained by the presence of the DOX/HACE nanoassembly released from DOX/HACE MS into the aqueous environment (Figure 2(C)). Interactions between HA and the CD44 receptor seem to contribute to the enhanced cellular accumulation (Cho et al., 2011, 2012; Jin et al., 2012). CD44 receptor-mediated endocytosis of HACE-based nanoparticles including DOX in CD44 receptor-positive cancer cells has already been demonstrated in our previous studies (Cho et al., 2011, 2012; Jin et al., 2012; Park et al., 2014; Lee et al., 2016, 2017; Jeong et al., 2017). The current data imply that more drugs can be internalized into the liver cancer cells in the DOX/HACE MS group, rather than DOX MS group, after the embolization of the hepatic artery.

### *In vitro* anticancer activities

The *in vitro* anticancer potentials of the developed DOX-loaded MSs were assessed by antiproliferation and apoptosis assays in HepG2 and McA-RH7777 cells (Figure 5(B,C)). In HepG2 cells (Figure 5(B)), the DOX/HACE MS group showed significantly higher antiproliferation efficacy compared to the DOX MS group in the range of 0.1–10 μg/mL DOX concentration (*p* < .05). The IC_50_ values of the DOX MS and DOX/HACE MS groups were 5.66 ± 0.08 μg/mL and 3.53 ± 0.05 μg/mL (*p* < .05), respectively. Cell viability values of blank MS and blank HACE MS were approximately 100%. This means that the antiproliferation potentials of DOX-loaded MSs were based on the pharmacologic actions of DOX and not by the toxicity of the components (i.e. PLGA, TIBA, and HACE) of MSs. Additionally, in McA-RH7777 cells (Figure 5(B)), the cell viability values of the DOX/HACE MS group were lower than those of the DOX MS group at the 0.5–10 μg/mL DOX concentration range (*p* < .05). The IC_50_ values of the DOX MS and DOX/HACE MS groups were 8.52 ± 0.28 μg/mL and 4.23 ± 0.12 μg/mL (*p* < .05), respectively. The cell viability values of blank MS and blank HACE MS were also approximately 100%, indicating negligible cytotoxicity of the ingredients of the MSs. Higher antiproliferation efficiency of the DOX/HACE MS group compared with DOX MS group in both liver cancer cells might be related to the release of the DOX/HACE-based nanoassembly in the aqueous environment and its receptor-mediated endocytosis.

Anticancer activities of the developed MSs in HepG2 and McA-RH7777 cells were also assessed by the apoptosis assay (Figure 5(C)). The Annexin V and PI-based assays were used for the evaluation of apoptotic events of DOX-loaded MSs. The population percentages in the lower right (LR) and upper right (UR) panels indicate early and late apoptosis, respectively. Therefore, the sum of population percentages in the LR and UR panels can be used for the estimation of apoptotic events in cancer cells. In HepG2 cells, the population percentages in the LR and UR panels of DOX, DOX MS, and DOX/HACE MS were 23.7 ± 0.9%, 33.9 ± 1.3%, and 37.2 ± 1.5%, respectively (Figure 5(C)). The apoptotic events in the DOX/HACE MS group were higher than those of the DOX and DOX MS groups (*p* < .05). In the case of McA-RH7777 cells, the population percentages in the LR and UR panels of DOX, DOX MS, and DOX/HACE MS were 15.3 ± 0.7%, 20.7 ± 0.3%, and 23.7 ± 0.1%, respectively (Figure 5(C)). The DOX/HACE MS group also exhibited higher apoptotic efficiency compared to the DOX and DOX MS groups in McA-RH7777 cells (*p* < .05). The apoptosis data may support for the improved antiproliferation potential of HACE/DOX MS group compared with the DOX and DOX MS groups, in both kinds of cells.

### *In vivo* antitumor efficacy

**Figure 6.**
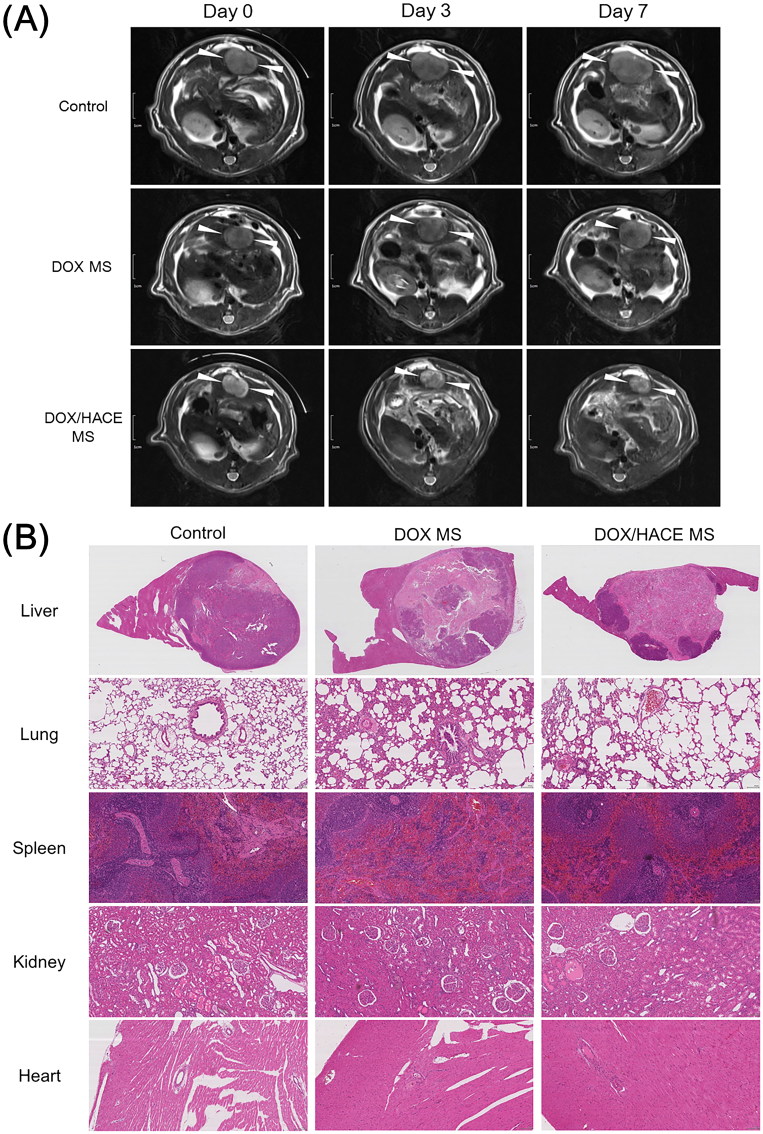
*In vivo* anticancer activities in the McA-RH7777 tumor-implanted rat model. (A) Serial MR images of liver tumors in control (sham operation), DOX MS, and DOX/HACE MS groups on day 0 (pre-treatment status), 3, and 7 after IA administration. White arrowheads in MR images indicate liver tumor. The length of scale bar in the left side is 1 cm. (B) H&E staining images of dissected liver and tumor, lung, spleen, kidney, and heart in control, DOX MS, and DOX/HACE MS groups. The length of scale bar (shown in the lower right side) in the images of lung, spleen, kidney, and heart is 100 μm.

The anticancer activities of the developed DOX MS and DOX/HACE MS were tested in McA-RH7777 tumor-implanted rat model (Figure 6). Tumor growth inhibitory effects of DOX-loaded MS formulations were evaluated by calculating tumor volume with MR imaging (Figure 6(A)). There was no significant difference in tumor volumes among experimental groups (control, DOX MS, and DOX/HACE MS) at day 0. Relative ratios of tumor volume in control and DOX MS groups at day 0/3/7 were 1.00/1.72/2.70 and 1.00/1.57/2.20, respectively. On the contrary, the declining pattern was observed in the relative ratio of tumor volume in DOX/HACE MS group (1.00/0.69/0.61 at day 0/3/7). Tumor growth suppression efficacy of DOX/HACE MS group was higher than those of control and DOX MS groups. Notably, tumor volume of DOX/HACE MS group was 24% of that of control group on day 7. After IA administration of DOX MS and DOX/HACE MS with control group (no treatment) in McA-RH7777 tumor-inoculated rats, liver tissues bearing the McA-RH7777 tumor were dissected on day 7 and they were stained by the H&E reagent (Figure 6(B)). Liver tumor can be discerned from normal liver tissue by round-shape boundary. Dark purple color and light purple color in the tumor region indicate viable tumor and necrosis portion, respectively. The viable tumor portions of the control (no treatment), DOX MS, and DOX/HACE MS treatments were 78%, 42%, and 25%, respectively. The apoptotic efficiency was tested by the TUNEL assay of dissected liver tissues (Figure S2). The brown color (by DAB staining) indicates the level of apoptotic cells in the dissected McA-RH7777 tumor-bearing liver tissues. The apoptotic area (brown color) of the DOX/HACE MS group was larger than that of the DOX MS group. The released DOX/HACE-based nanoassembly from MSs in the hepatic artery was likely internalized via receptor-mediated endocytosis. Enhanced antiproliferation and apoptosis efficacies of DOX/HACE MS in McA-RH7777 cells can explain the *in vivo* results (Figure 5). Toxicity of administered DOX-loaded MSs was assessed by H&E staining of lung, spleen, kidney, and heart (Figure 6(B)). Compared to the images of control (no treatment) group, no obvious alteration was presented in DOX MS and DOX/HACE MS-administered groups in histological staining data of the lung, spleen, kidney, and heart. MSs administered via an IA route seem to provide primarily locoregional anticancer effects rather than unspecified toxicities in other organs. We conclude that DOX/HACE composite-incorporated MS may show superior anticancer activities than DOX MS after IA administration during TACE therapy of liver cancer.

## Conclusions

DOX/HACE-based nanoassembly-releasing MSs were fabricated for TACE therapy of liver cancer. The DOX/HACE composite was loaded into PLGA-based MS for improved anticancer activities via CD44 receptor-mediated endocytosis of the DOX/HACE-based nanoassembly. DOX/HACE MS with a 27 μm mean diameter and spherical shape was fabricated by a modified emulsification method described in this study. Compared with the DOX MS group, the DOX/HACE MS group exhibited a more sustained drug release at pH 7.4. In HepG2 and McA-RH7777 cells, the DOX/HACE MS group showed higher cellular accumulation efficiency of DOX than the DOX MS group. Antiproliferation and apoptosis efficacies of the DOX/HACE MS group were significantly higher than those of the DOX MS group in both HepG2 and McA-RH7777 cells. After IA administration in a McA-RH7777 tumor-implanted rat model, the DOX/HACE MS group exhibited efficient suppression of tumor growth and higher apoptotic events compared with the DOX MS group. The release of the DOX/HACE-based nanoassembly and its efficient cellular internalization by receptor-mediated endocytosis likely contributed to the improved anticancer activities of DOX/HACE MS (compared to DOX MS) against liver cancers in TACE therapy.

## Supplementary Material

SI_R1.docx
