# Supplementary material for: Hyaluronic acid/doxorubicin nanoassembly-releasing microspheres for the transarterial chemoembolization of a liver tumor
Source: Drug Deliv. 2018 Jun 17;25(1):1472–83. doi: 10.1080/10717544.2018.1480673 (PMC6058484; doi:10.1080/10717544.2018.1480673)
Supplement: SI_R1.docx [file IDRD_A_1480673_SM7688.docx]

**Supporting Information**

**Hyaluronic acid/doxorubicin nanoassembly-releasing microspheres for the transarterial chemoembolization of a liver tumor**

Song Yi Lee^a,1^, Jin Woo Choi^b,1^, Jae-Young Lee^c^, Dae-Duk Kim^d^, Hyo-Cheol Kim^b,*^, Hyun-Jong Cho^a,**^

^a^College of Pharmacy, Kangwon National University, Chuncheon, Gangwon 24341, Republic of Korea; ^b^Department of Radiology, Seoul National University Hospital, Seoul National University College of Medicine, Seoul 03080, Republic of Korea; ^c^College of Pharmacy, Chungnam National University, Daejeon 34134, Republic of Korea; ^d^College of Pharmacy and Research Institute of Pharmaceutical Sciences, Seoul National University, Seoul 08826, Republic of Korea.

^1^ These authors equally contributed to this work.

^*^ Corresponding author. Tel.: +82 2 2072 2584; fax: +82 2 743 6385

**Corresponding author. Tel.: +82 33 250 6916; fax: +82 33 259 5631.

*E-mail addresses*: angiointervention@gmail.com (H.-C. Kim), hjcho@kangwon.ac.kr (H.-J. Cho).





**Figure S1.** Chemical structure of HACE.


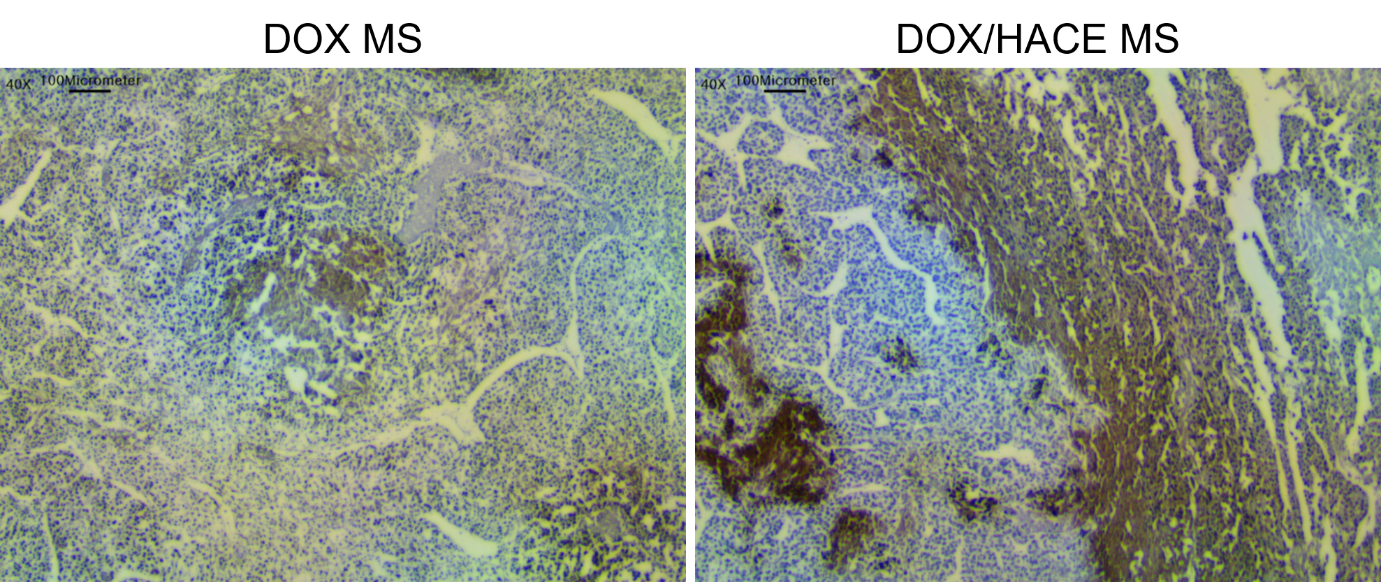


**Figure S2.** TUNEL assay image of DOX MS and DOX/HACE MS-treated groups. The length of bar is 100 μm.

**Table S1**

Particle characterization of DOX-loaded MSs.

| **Formulation** | **Mean diameter (μm)** | **Encapsulation efficiency (%)**^a^ |
| --- | --- | --- |
| DOX MS | 25 ± 3 | 97.0 ± 0.4 |
| DOX/HACE MS | 27 ± 4 | 65.1 ± 5.0 |

Mean diameter is presented in the respective of volume.

Data are expressed as mean ± SD (*n* = 3).

^a^$\text{Εncapsulation efficiency (\%)}=\frac{\text{actual amount of DOX in MS}}{\text{theoretical amount of DOX in MS}}\times100$
